# Evolution of sex-ratio: Brief review with mathematical study of some simple novel models

**DOI:** 10.1590/1678-4685-GMB-2021-0053

**Published:** 2021-07-26

**Authors:** Paulo A. Otto

**Affiliations:** 1 Universidade de São Paulo, Instituto de Biociências, Departamento de Genética, Laboratório de Genética Humana, São Paulo, SP, Brazil.

**Keywords:** Sex-ratio (s.r.), evolutionary stable s.r., extraordinary s.r., population genetic models

## Abstract

Besides reviewing the unusual case of sex-ratio in the lemming and presenting alternative analyses of general models in which the shift in the usual sex-ratio 1:1 is determined by autosomal or sex-linked mutant alleles, three novel models are presented, in which the shift on the progeny sex-ratio depends on the number of copies of a mutant allele present in the parental pair. The analysis of these models with additive effects shows that: 1) autosomal mutations that alter the usual sex**-**ratio are eliminated from the population; 2) mutations occurring on the X chromosome lead to an evolutionary stable **1:1** sex-ratio only if the mutation favors the production of males; when the mutant allele favors the production of females, however, females will prevail in the population, with a frequency dependent impact on δ (the deviation from the usual 0.5 proportion) ; for most of the range of possible values of δ the stable but extraordinary sex-ratio will vary from **1 male : 1 female** to **1 male : 3 females** or **1 male : 2 females** approximately depending whether the mutant allele is randomly inactivated or not.

## Introduction

The main purpose of this paper is to deal with the problem of sex-ratio determination among mammalians in general, that is, with a group where primary sex determination depends exclusively on a simple symmetric chromosomal mechanism XX-XY, purposefully avoiding complicated mechanisms of sex-ratio control and sex determination that are fairly common in other groups of animals (as in the case, for example, of many insects). The text starts with this general introduction, reviewing the issue on its basic aspects; the section is then followed by the analysis of six different models (numbered 1 to 6), which are grouped together at the end of the paper, using elementary mathematical methods usually within the grasp of biologists and geneticists interested in general issues outside their specialized area of professional expertise. 

Three out of the six models (numbered as 2, 5 and 6) assume that the shift on the progeny sex-ratio is dependent on the number of copies of a mutant allele present in the parental pair. Model 2 assumes that the sex-ratio is dependent on an autosomal mutant allele; models 5 and 6 deal with one of such alleles located on the X-chromosome, taking into account or not the process of random inactivation of the corresponding locus. 

The analytical methods in all models are (at least partly) original in spite of their flagrant simplicity. Only models numbered as 2, 5 and 6 are novel, in spite of producing results that at least partly coincide with some important or basic results already widely reported in the literature on the subject. So, this paper should be considered a review article in spite of its sparse humble novelties and contributions. 

The intuitive argumentation summarized in the next paragraph, showing that the **1 male : 1 female** sex-ratio is evolutionary stable, is simple but subtle since it requires the analysis of two generations. During many years the reasoning was attributed to Fisher (1930), but Edwards (1998) showed that the idea was actually fathered by other authors in the middle and late years of the 19th century, among them Carl Düsing (1983, 1884), a German biologist.

Let us suppose that in a given population for any reason there exists a surplus of males. Since each individual results from a fertilization in which the two gametes equally participate, females will have on average a larger offspring number than males. The individuals from this population that have a proportion of female progeny larger than the average frequency of females will have on average more grandchildren than the rest of the population, and this will play down the existing excess of males. The inverse argumentation (a population where initially there exists more females than males) is identical, that is, individuals from this population that have a proportion of male progeny larger than the average frequency of males will have on average more grandchildren than the rest of the population, and this will play down the existing excess of females.

If the tendency to produce a progeny sex-ratio outside the usual ratio **1:1** is hereditary, at equilibrium a sex-ratio of approximately **1 male :1 female** is expected: let us suppose, for example, that the factor responsible for an excess of males is a mutation that makes its carriers produce more males than females. Then the individuals that don't carry this mutation will produce on average more females (individuals that on average have a larger offspring number); therefore these individuals will have on average more grandchildren and the wild-type allele, responsible for the usual **1 male : 1 female** sex-ratio (and that in this case represents a allele producing an excess of females while any excess of males persists) will tend to increase in frequency. The inverse argumentation is identical, that is, in populations where the mutation effect is to produce an excess of females, alleles responsible for the usual **1 male : 1 female** sex-ratio (in this case producing an excess of males) tend to increase in frequency. We conclude therefore that the **1 male : 1 female** sex-ratio is evolutionary stable.

The subject is mathematically attractive and has received many theoretical contributions, listed or discussed in good reviews like the one by Charnov (1982). Following the reasoning developed in the previous paragraph and the ideas of Fisher (1930) on parental expenditure, many authors, using different models, showed that the sex-ratio is evolutionary stable when autosomal mutations that produce a shift on the **1 male : 1 female** sex-ratio are carried by the individuals or their parents (Scudo, 1946; Shaw and Mohler, 1953; Shaw, 1958; Bodmer and Edwards, 1960; Nur, 1974; Uyenoyama and Bengtsson, 1979). The mathematical level of most of these papers is generally beyond the grasp of the usual biologist, but in any case the reading of these excellent papers (as well as the many others cited in this review) is especially recommended to anyone interested on the issue of sex-ratio. Fairly good descriptions and analyses in a more accessible level of mathematics without significant loss of rigor are found in the textbooks of Crow and Kimura (1970) and Maynard-Smith (1989). 

**Figure 1 -:**
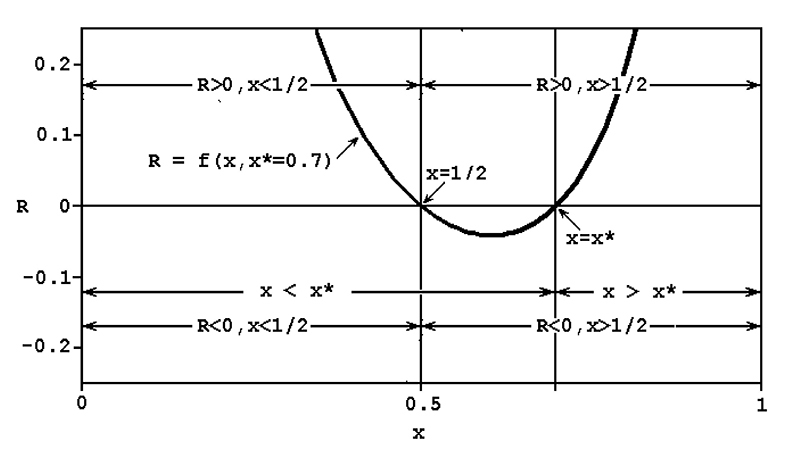
Behavior of the function R = (1-2x)(x*-x)/[2x(1-x)], where x and 1-x are the usual proportions of males and females respectively and x* is the proportion of males determined by a mutant autosomal allele (please consult the paragraph above for other details).

The simplified general mathematical argumentation detailed below follows the reasoning of the papers above and was adopted from Maynard-Smith (1989), who considered the rare autosomal allele **M**, with no expression in males but that makes females produce m* sons and f* daughters, contrarily to the offspring of other individuals (carriers of the wild-type allele **+**), that have **m** sons and **f** daughters. As pointed out by this and other authors as well, it is important to stress that the presence of the allele **M** produces only a shift or distortion in the sex-ratio, so that m + f = m* + f*. Letting **P** and **p** be the frequencies of **M/+** females and **M/+** males and considering the frequencies of **M/M** males and females as negligible, from the possible crossings between females and males (**M/+ × +/+**, **+/+ × M/+** and **+/+ × +/+**) a total of P' = f*P/2f + p/2 and p' = m*P/2m + p/2 heterozygous **M/+** females and males respectively will exist in the following generation. So it comes out that P'+p' = p + P(fm*+mf*)/2mf = p + P + P(fm*+mf*-2mf)/2mf = p + P + RP, where R = (fm*+mf*-2mf)/2mf = (1-2x)(x*-x)/[2x(1-x)], where **x = proportion of males** and **1-x = proportion of females**. **P'+p'** will be larger than **P+p** if **R > 0**. **R** is larger than zero if x < 1/2 and x* > x or if x > 1/2 and x > x*. Therefore, when **x < 1/2,** the mutants that increase the sex-ratio increase their frequency; and when **x > 1/2** the mutants that decrease the sex-ratio increase their frequency. Therefore, the solution of the equation **R = 0 (x = 1/2)**, is an evolutionary stable sex-ratio (Figure 1).

This general issue is analyzed in detail by an alternative method (**Model 1: Sex-ratio disruption promoted by an autosomal mutant allele A present in the parental pair**) and also revisited under a novel perspective (Model 2: **Autosomal allele A, without sex limitation, that alters the progeny sex-ratio according to the number of mutant allele copies present in the parental pair**); these two models are presented at the end of this introduction, together with four other models, in the section **Analysis of some sex-ratio models**.

The argument discussed above can be easily generalized for the generic situation of parental expenditure suggested by Fisher (1930). Using the same symbols as before and adopting again the reasoning in Maynard-Smith (1989), let m + kf = m* + kf* = C, where **C** represents the total expenditure in the offspring, a female costing **k** times more than a male. From these two equations we obtain **f = (C-m)/k** and f* = (C-m*)/k. Replacing these values in R = (f*/f + m*/m)/2 - 1, we immediately obtain R = (C-2m)(m*-m)/[2m(1-m)]. 

R will be greater than zero if m < C/2 and m* > m or if m > C/2 and m > m*. Therefore, if m < C/2, mutants that increase the value of m will tend to increase their frequency; if m > C/2, mutants that decrease the value of m will tend to increase their frequency. Therefore the evolutionary stable sex-ratio is given by m = C/2. If k = 1, C = = m* + f* and x = 1/2 (as seen before).

It has been shown, however, that the **1 male : 1 female** prediction may fail when sex-linked (either on the X or in the Y chromosome) factors influencing the sex-ratio are considered; this case of extraordinary sex-ratios was also the subject of several in-depth papers (Hamilton, 1967; Thomson and Feldman, 1975; Uyenoyama and Bengtsson, 1981; Eshel and Feldman, 1982). The models used are in general more complicated, in addition to having a large number of restrictive assumptions and, in general, making use of fancier methods of mathematics.

In the case of a mutant allele located on the Y chromosome the reason for an unusual sex-ratio is very simple, because (1) the grandchild progeny number from daughters has no association at all with the allele carried on the Y chromosome by their fathers; and (2), if the mutant determines a larger proportion of male progeny, it will compete freely with its normal allele, eventually replacing it. When the deviation is relatively large from the usual 1:1 sex-ratio, the population itself will become extinct due to the lack of a minimally feasible number of female individuals, as pointed out by many authors (e.g., Hamilton, 1967).

The case of the mutant allele located in the Y chromosome is analyzed in detail, together with other models presented, as afore-mentioned, at the end of this introduction (**Model 3: Sex-ratio disruption promoted by a mutant allele A present in the Y chromosome**).

In the case of a mutation carried on the X chromosome the situation is more complicated because heterozygous females transmit the allele with equal probabilities to all their children irrespective of their sex, but hemizygous male carriers transmit the allele to all their female progeny but to none of their male progeny. This fact disrupts to a certain degree the clear grandparent-grandchild association that is intuitively obvious in the autosomal case. When an X-linked mutation inducing the production of a surplus of males is expressed only in the spermatozoa from these individuals a catastrophic population fate similar to what takes place in the Y mutant case is expected, as shown by Hamilton (1967) using intuitive argumentation and simulation studies. In some instances, however, the result is not so drastic, as is the case of the wood lemming, a small rodent from the Arctic tundra, whose populations are characterized by drastic size number fluctuations and by a conspicuous excess of females. This special case of unusual sex-ratio, whose dynamics is already known in the literature, is also analyzed in detail in the model section at the end of this introduction (**Model 4: Sex-ratio disruption in lemming populations**), together with two additional novel models dealing with X-linked mutant alleles determining a shift in the usual 1:1 sex-ratio under the perspective that the progeny sex-ratio depends on the number of mutant alleles present in the parental pair (**Models 5 and 6: X-linked mutant allele located in active or in randomly inactivated loci respectively).**

## Analysis of some sex-ratio models

### Model 1: Sex-ratio disruption promoted by an autosomal mutant allele A present in the parental pair

We start by considering, like Maynard-Smith did, the population proportion of males as **x** (corresponding to a sex-ratio of **x/(1-x) : 1**) and **x + δ**_1_ (such that **1-x > |δ**_1_ **| > 0**) the proportion of males in the progeny of male or female carriers of an autosomal mutant allele **A**, that exhibits (unlike the example used by Maynard-Smith) no sex limitation. Since **x = 1/2 + δ**_2_ (such that **1/2 > |δ**_2_ **| > 0**), the model analysis becomes much more direct and simplified if we use **x + δ**_1_ **= 1/2 + δ**_2_ **+ δ**_1_ **= 1/2 + δ** (such that **1/2 > |δ| > 0**) instead of **x + δ**_1_. Let the frequency of the mutant **A** allele be **q**_t_. Since this frequency is (at least initially) very small, because the mutant allele is introduced by mutation, population frequencies of **AA**, **Aa** and **aa** individuals can be taken respectively as **P**_t_ **(AA) = q**_t_^2^ **≈ 0**, **P**_t_ **(Aa) = 2p**_t_ **q**_t_ **≈ 2q**_t_ and **P**_t_ **(aa) = (1-q**_t_ **)** ^2^ **≈ 1-2q**_t_. Since random mating is expected to occur, the frequencies of the various crossings are:

P_t_(Aa × Aa) = 4q_t_^2^ ≈ 0, 

P_t_(Aa × aa) = 4q_t_(1-2q_t_) ≈ 4q_t_, 

and 

P_t_(aa × aa) = (1-2q_t_)^2^ ≈ 1-4q_t_.

The expected progeny frequencies of heterozygous (**Aa**) and homozygous (**aa**) males and females are respectively

m_t+1_(Aa) = 2q_t_(1/2+δ) , 

m_t+1_(aa) = 2q_t_(1/2+δ)+(1-4q_t_)/2 , 

and

f_t+1_(Aa) = 2q_t_(1/2-δ) ,

f_t+1_(aa) = 2q_t_(1/2-δ)+(1-4q_t_)/2 ;

from these quantities we derive the population frequencies of males and females in the offspring generation, which are respectively

m_t+1_(Aa)+m_t+1_(aa) = m_t+1_ = 1/2 + 4q_t_δ 

and 

f_t+1_(Aa)+f_t+1_(aa) = f_t+1_ = 1/2 - 4q_t_δ .

We notice that genotype frequencies did not change in the whole offspring population, because **m**_t+1_ **(Aa)+f**_t+1_ **(Aa) = 2q** and **m**_t+1_ **(aa)+f**_t+1_ **(aa) = 1-2q ,** as in the previous generation**;** however, the genotypes occur now with different frequencies among males and females:

Pm_t+1_(Aa) = m_t+1_(Aa)/m_t+1_ = q_t_(1+2δ)/(1/2+4q_t_δ)

Pm_t+1_(aa) = m_t+1_(aa)/m_t+1_ = (1/2-q_t_+2q_t_δ)/(1/2+4q_t_δ)

and

f_t+1_(Aa) = f_t+1_(Aa)/f_t+1_ = q_t_(1-2δ)/(1/2-4q_t_δ)

f_t+1_(aa) = f_t+1_(aa)/f_t+1_ = (1/2-q_t_-2q_t_δ)/(1/2-4q_t_δ) ,

so that the frequencies of the **A** allele among males and females are respectively

Pm_t+1_(A) = Pm_t+1_(Aa)/2 = q_t_(1/2+δ)/(1/2+4q_t_δ)

and 

Pf_t+1_(A) = Pf_t+1_(Aa)/2 = q_t_(1/2-δ)/(1/2-4q_t_δ).

Since each individual results from the fertilization of a female gamete by a male one, independently from the sex-ratio prevailing in the population, the next generation frequency **q’** of allele **A** in the whole population is given by

q' = [Pm_t+1_(A)+ Pf_t+1_(A)]/2 

 = q_t_[(1/2+δ)/(1/2+4q_t_δ)+(1/2-δ)/(1/2-4q_t_δ)]/2

 = q_t_(1-16q_t_δ^2^)/(1-64q_t_^2^δ^2^) ≈ q_t_ - 16q_t_^2^δ^2^ = q_t_ - (4q_t_δ)^2^ .

Since **1/2 > |δ| > 0**, it comes out that **q’** is always smaller than **q**_t_, so that any autosomal mutant allele that disrupts the usual **1:1** sex-ratio is always eliminated from the population.

### Model 2: Autosomal allele A, without sex limitation, that alters the progeny sex-ratio according to the number of mutant allele copies present in the parental pair

In the offspring of **aa × aa** crossings the frequency of males and females is **0.5**, that corresponds to an **1 male : 1 female** sex-ratio. The offspring of other possible crossings has a proportion of males and females respectively higher and smaller than 0.5, proportionally to the number of **A** genes present in the parental pair. We shall have, therefore, five offspring types with different proportions of males and females (Table 1), in which **q**_t_ and **p**_t_ are the frequencies of alleles **A** and **a** in generation **t** .

**Table 1 -:** Population frequencies of crossing types and progeny sex proportions (model 2). A is an autosomal mutant allele responsible for a distortion in the sex-ratio depending on the number of copies present in the parental pair.

crossing types	crossing freq.	male proportion	female proportion
aa × aa	p_t_^4^	0.5	0.5
aa × Aa	4p_t_^3^q_t_	0.5 + δ	0.5 - δ
aa × AA	2p_t_^2^q_t_^2^	0.5 + 2δ	0.5 - 2δ
Aa x Aa	4p_t_^2^q_t_^2^	0.5 + 2δ	0.5 - 2δ
AA × Aa	4p_t_q_t_^3^	0.5 + 3δ	0.5 - 3δ
AA × AA	q_t_^4^	0.5 + 4δ	0.5 - 4δ

If we define, as above, **δ** as a positive quantity, its domain is obviously **0 ≤ δ ≤ 1/8**. But of course **δ** can be a negative quantity as well: in this case, its domain is **-1/8 ≤ δ ≤ 0**. In fact, when **δ = -1/8**, the five possible offspring male proportions listed on the table above take values 1/2, 3/8, 1/4, 1/8, and 0, respectively; when **δ = 1/8**, the corresponding values are 1/2, 5/8, 3/4, 7/8, and 1.

The proportions **m**_t+1_ **(aa)**, **m**_t+1_ **(Aa)** and **m**_t+1_ **(AA)** of **aa**, **Aa** and **AA** males as well as the population frequency of males **m**_t+1_ **= m**_t+1_ **(aa) + m**_t+1_ **(Aa) + m**_t+1_ **(AA)** and the corresponding proportions of females **f**_t+1_ **(aa)**, **f**_t+1_ **(Aa), f**_t+1_ **(AA),** and **f**_t+1_ **= f**_t+1_ **(aa) + f**_t+1_ **(Aa) + f**_t+1_ **(AA)** in the offspring generation (**t+1**) are straightforwardly taken from the table above, after weighing the possible offspring proportions of males (**0.5**, **0.5+δ,** ..., **0.5+4δ**) and females (**0.5**, **0.5-δ,** ..., **0.5-4δ**) from each crossing type by their corresponding parental mating frequencies **p**_t_^4^, ..., **q**_t_^4^ (Table 2), so that the frequencies of **aa**, **Aa** and **AA** individuals among males of generation **t+1** are:

Pm_t+1_(aa) = m_t+1_(aa)/m_t+1_ = p_t_^2^.(1/2+2δq_t_)/(1/2+4δq_t_)

Pm_t+1_(Aa) = m_t+1_(Aa)/m_t+1_ = 2p_t_q_t_.[1/2+2δ(1/2+q_t_)]/(1/2+4δq_t_)

Pm_t+1_(AA) = m_t+1_(AA)/m_t+1_ = q_t_^2^.[1/2+2δ(1+q_t_)]/(1/2+4δq_t_) .

**Table 2 -:** Progeny AA, Aa and aa genotype frequencies among males and females after one generation of random crossings (model 2).

genotypes	males	females	total
aa	p_t_^2^.(1/2+2δq_t_)	p_t_^2^.(1/2-2δq_t_)	p_t_^2^
Aa	2p_t_q_t_[1/2+2δ(1/2+q_t_)]	2p_t_q_t_[1/2-2δ(1/2+q_t_)]	2p_t_q_t_
AA	q_t_^2^.[1/2+2δ(1+q_t_)]	q_t_^2^.[1/2-2δ(1+q_t_)]	q_t_^2^
total	1/2 + 4δq_t_	1/2 - 4δq_t_	1

An identical procedure is used to obtain the corresponding frequencies of **aa**, **Aa** and **AA** individuals among females of this same generation **t+1**: 

Pf_t+1_(aa) = p_t_^2^.(1/2-2δq_t_)/(1/2-4δq_t_)

Pf_t+1_(Aa) = 2p_t_q_t_.[1/2-2δ(1/2+q_t_)]/(1/2-4δq_t_)

Pf_t+1_(AA) = q_t_^2^.[1/2-2δ(1+q_t_)]/(1/2-4δq_t_) .

The allele **A** frequencies **Pm**_t+1_ **(A)** and **Pf**_t+1_ **(A)** among males and females are straightforwardly obtained from the genotype frequencies above, taking values:

Pm_t+1_(A) = Pm_t+1_(AA) + Pm_t+1_(Aa)/2 

 = [q_t_.(1/2 + 3δq_t_) + δq_t_]/(1/2 + 4δq_t_)

Pf_t+1_(A) = Pf_t+1_(AA) + Pf_t+1_(Aa)/2 

 = [q_t_.(1/2 - 3δq_t_) - δq_t_]/(1/2 - 4δq_t_) .

Since each individual results from the fertilization of a female gamete by a male one, independently from the sex-ratio prevailing in the population, the next generation frequency **q**_t+2_ = **q’** of allele **A** in the whole population is given by

q’ = [Pm_t+1_(A)+Pf_t+1_(A)]/2 = q_t_(1/4-12δ^2^q_t_^2^-4δ^2^q_t_)/(1/4-16δ^2^q_t_^2^)

 = q_t_ - 4δ^2^q_t_^2^(1-q_t_)/(1/4-16δ^2^q_t_^2^).

The inspection of this formula shows clearly that **q’ < q**_t_ , given the obvious restrictions **1 > q**_t_ **- 4δ** ^2^ **q**_t_^2^ **(1-q**_t_ **)/(1/4-16δ** ^2^ **q**_t_^2^ **) > 0** , **1 > q**_t_ **> 0** , and **1/8 > |δ| > 0**; therefore, at equilibrium, that is when **t** tends to infinity, **q = 0**, that is, ***any mutation that alters the sex-ratio 1 male : 1 female (or the proportion c = 1/2 of males) is eliminated from the population, irrespective whether δ is larger or smaller than zero*** .

The frequency of the autosomal mutant **A** allele is always very small, because the allele is not only introduced by mutation but strongly selected as well. Then the frequencies of **aa**, **Aa**, and **AA** individuals can be approximated as **(1-q**_t_^2^ **) ≈ 1-2q**_t_, **2p**_t_ **q**_t_ **≈ 2q**_t_, and **q**_t_^2^ **≈ 0**, and the only possible population crossings are **aa × aa** and **aa × Aa**, that will take place with corresponding probabilities **(1-2q**_t_ **)** ^2^ **≈ 1-4q**_t_ and **4q**_t_. Under this approximation, the model just detailed coincides almost exactly with the generalized one derived before (**model 1**), with the final result **q’ = q**_t_ **- 16q**_t_^2^ **δ** ^2^ **2 = q**_t_ **- (4q**_t_ **δ)** ^2^ (a result that can also be obtained straightforwardly from the complete formula just derived by neglecting most non-linear terms of **q**_t_).

### Model 3: Sex-ratio disruption promoted by a mutant allele A present in the Y chromosome

**Figure 2 -:**
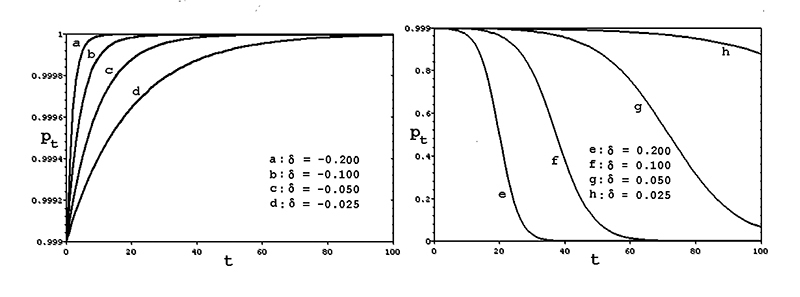
Evolution of the wild type allele that determines the usual male population proportion; when the mutant allele on the Y chromosome determines a male progeny frequency larger than c, the normal allele is eliminated from the population; when the mutant allele produces a male progeny smaller than c, the mutant allele is eliminated so that the c population frequency of males is restored (model 3).

The relatively simple model detailed in the lines below explains the behavior of the allele **a**, situated on the Y chromosome, that determines, in the offspring of their carriers, a proportion of males **c = 1/2**; its mutant allele **A** determines a proportion of males **s = c + δ = 1/2 + δ**. Letting **p**_t_ and **q**_t_ **= 1 - p**_t_ be the frequencies, in generation **t**, of alleles **a** and **A**, the following recurrence relation is obtained: **p**_t+1_ **= p**_t_ **/[1 + 2δ(1-p**_t_ **)]**. If **δ < 0, p**_t_ **/[1 + 2δ(1-p**_t_ **)] > p**_t_ and therefore **p**_t+1_ **> p**_t_ so that the limit of **p**_t_ as **t** tends to infinity is one; the mutant allele **A** will then be completely eliminated from the population and the proportion of males in the entire population will be given by **c = 1/2**. Conversely, if **δ > 0, p**_t_ **/[1 + 2δ(1-p**_t_ **)] < p**_t_ and therefore **p**_t+1_ **< p**_t_ so that the limit of **p**_t_ as **t** tends to infinity would be zero and the mutant allele **A** would be fixed in the population, whose proportion of males will then be given by **s = c + δ = 1/2 + δ**. The same results are obtained when one considers the general solution **p**_t_ **= p**_0_ **/[p**_0_ **+ (1-p**_0_ **)(1+2δ)** ^t^ **]** of the fractional difference equation of first order **p**_t+1_ **= p**_t_ **/[1 + 2δ(1-p**_t_ **)]**: taking the limit of the general expression as **t** tends to infinity we obtain straightforwardly the equilibrium values **{p = 1, q = 0}** when **δ < 0** or **{p = 0, q = 1}** when **δ > 0**. Figure 2 shows the evolution of the frequency **p**_0_ **→ p**_100_ of the wild-type allele **a** as function of several negative (-1/5 , ... , -1/40) and positive (1/5 , ... , 1/40) values of **δ**, for the hypothetical case **p**_0_ **= 0.999** (when the initial frequency **q**_0_ of the mutant allele **A** is 1/1000). 

The main conclusion from the simplified analysis shown above is that the **1 male : 1 female** ratio is attained at equilibrium only if the mutant **A** allele on the Y chromosome determines an excess of females. When this allele shifts the **1 male : 1 female** ratio favoring the production of males, however, stable proportions of **c + δ = 0.5 + δ** and **c - δ = 0.5 - δ** of males and females respectively will be expected at equilibrium. This situation (**δ > 0**) represents an important case in which the **1 male : 1 female** prediction fails. As already suggested by several authors, the extinction of the population (that for large values of **δ** could eventually take place on the long range of time when all individuals produced in the population would be males) would be prevented by the occurrence of independent autosomal mutations that would revert the situation, shifting the population eventually to an approximate **1 male : 1 female** stable ratio again.

### Model 4: Sex-ratio disruption in lemming populations (Bengtsson, 1977; Fredga et al., 1977)

Unlike all other five models presented here and that depend on autosomal or sex-linked alleles disrupting the usual 1:1 sex-ratio, the lemming case is somewhat different, since the sex-ratio shift in populations of this rodent is determined by a mutant chromosome **X'** whose effect is to produce a complete sex reversal on individuals **X'Y**, who are phenotypically females, like **XX** and **X'X** individuals. As a result, the male phenotype is associated only to the **XY** constitution. **XX** and **XY** individuals produce **X** and **Y** gametes in the usual way; **X'Y** individuals**,** besides being phenotypically females, produce only **X'** gametes (complete segregation distortion); and **X'X** individuals produce **X'** and **X** gametes with equal chances (Bengtsson, 1977; Fredga et al., 1977). If matings occur randomly and we let **a**_0_, **b**_0_, **c**_0_ and **d**_0_ be the initial population frequencies of **XY**, **X'Y**, **XX**, and **X'X** individuals respectively, the frequencies of these genotypes after a single generation of random crossings are respectively **a**_1_ **= c**_1_ **= (2c**_0_ **+d**_0_ **)/4(b**_0_ **+c**_0_ **+d**_0_ **)**, and **b**_1_ **= d**_1_ **= (2b**_0_ **+d**_0_ **)/[4(b**_0_ **+c**_0_ **+d**_0_ **)]**. The general recurrence equation for **b**_t_ is given by **b**_t+1_ **= (2b**_t_ **+d**_t_ **)/[4(b**_t_ **+c**_t_ **+d**_t_ **)]**. Since **c**_1_ **= a**_1_ **, d**_1_ **= b**_1_ and **a**_1_ **+ b**_1_ **= c**_1_ **+ d**_1_ **= 1/2**, this formula can be rewritten as **b**_t+1_ **= 3b**_t_ **/(4b**_t_ **+ 2)**, that is valid for **t ≥ 1.** At equilibrium, **b = 3b/(4b+2)**, **4b+2 = 3** and therefore **a = b = c = d = 1/4**, that is, all genotypes occur with equal population frequencies but there will be three times more females than males, independently from the initial frequencies of the four types **XY**, **X'Y**, **XX**, **X'X**.

### Model 5: allele A, X-linked, that alters the sex-ratio, and that is located in a locus that is active in both X chromosomes in females

Similarly to the autosomal model, the offspring of the possible crossings at generation **t+1** has a proportion of males higher than 0.5, proportional to the number of **A** genes present in the parental pair. We shall have, therefore, six offspring types with a total of four different proportions of males and females (Table 3).

**Table 3 -:** Population frequencies of crossing types and progeny sex proportions. A is an X-linked mutant allele responsible for a distortion in the sex-ratio depending on the number of copies present in the parental pair. It is assumed that the (A, a) locus is active in both X chromosomes among females (model 5).

crossing types	crossing freq.	male proportion	female proportion
aa × a	P_t_(a).P_t_(aa)	0.5	0.5
aa × A	P_t_(A).P_t_(aa)	0.5 + δ	0.5 - δ
Aa × a	P_t_(a).P_t_(Aa)	0.5 + δ	0.5 - δ
Aa x A	P_t_(A).P_t_(Aa)	0.5 + 2δ	0.5 - 2δ
AA × a	P_t_(a).P_t_(AA)	0.5 + 2δ	0.5 - 2δ
AA × A	P_t_(A).P_t_(AA)	0.5 + 3δ	0.5 - 3δ

If we define, as above, **δ** as a positive quantity, its domain is obviously **0 ≤ δ ≤ 1/6**. But of course **δ** can be a negative quantity as well: in this case, its domain is **-1/6 ≤ δ ≤ 0**. In fact, when **δ = -1/6**, the four possible offspring male proportions listed on the table above take values 1/2, 1/3, 1/6, and 0, respectively; when **δ = 1/6**, the corresponding values are 1/2, 2/3, 5/6, and 1.

If we depart from an initial equilibrium population, assuming that genotype frequencies do not differ significantly from HW proportions, that is, **P**_t_ **(a) ≈ p**_t_, **P**_t_ **(A) ≈ q**_t_, **P**_t_ **(aa) ≈ p**_t_^2^, **P**_t_ **(Aa) ≈ 2p**_t_ **q**_t_, and **P**_t_ **(AA) ≈ q**_t_^2^, and proceeding as in the autosomal case, we obtain the following male and female genotype frequencies at generation **t+1**:

Pm_t+1_(a) = m_t+1_(a)/m_t+1_ = (p_t_/2+2δp_t_q_t_)/(1/2+3δq_t_)

Pm_t+1_(A) = m_t+1_(A)/m_t+1_ = (q_t_/2+δp_t_q_t_+3δq_t_^2^)/(1/2+3δq_t_)

Pf_t+1_(aa) = f_t+1_(aa)/f_t+1_ = (p_t_^2^/2-δp_t_^2^q_t_)/(1/2-3δq_t_)

Pf_t+1_(Aa) = f_t+1_(Aa)/f_t+1_ = (2p_t_q_t_/2-2δp_t_q_t_-2δp_t_q_t_^2^)/(1/2-3δq_t_)

Pf_t+1_(AA) = f_t+1_(AA)/f_t+1_ = (q_t_^2^/2-2δp_t_q_t_^2^-3δq_t_^3^)/(1/2-3δq_t_).

From these quantities we calculate the frequencies **Pm**_t+1_ **(A)** and **Pf**_t+1_ **(A)** of the **A** allele among males and females:

Pm_t+1_(A) = m_t+1_(A)/m_t+1_ = (q_t_/2 + δp_t_q_t_ + 3δq_t_^2^)/(1/2 + 3δq_t_)

Pf_t+1_(A) = Pf_t+1_(AA) + Pf_t+1_(Aa)/2 = (q_t_/2 - δp_t_q_t_ - 3δq_t_^2^)/(1/2 - 3δq_t_)

so that the next generation frequency **q**_t+2_ = **q’** of the **A** allele in the whole population will be given by

q’ = [Pm_t+1_(A) + 2Pf_t+1_(A)]/3

 = q_t_ - 2δ(1-q_t_)q_t_(1-18δq_t_)/[3(1-36δ^2^q_t_^2^)].

**Figure 3 -:**
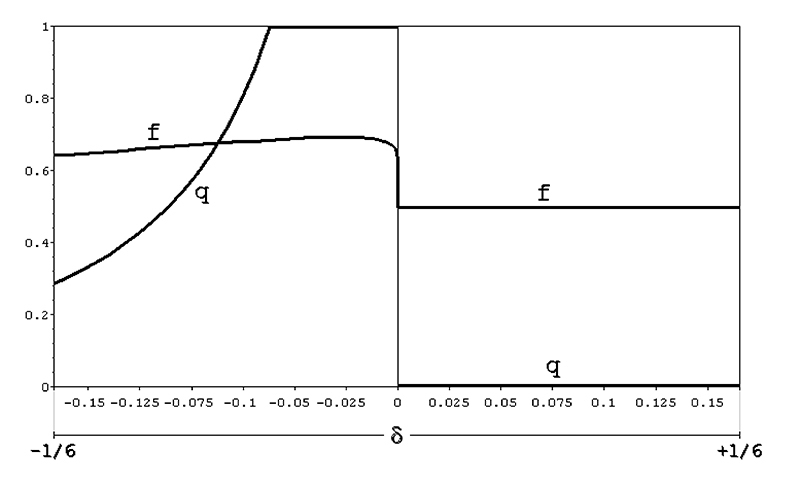
Population equilibrium frequencies of the mutant X-linked allele A (q) and of corresponding female frequencies (f), according to the value of the distortion factor **δ (model 5).**

Since **-1/6 < δ < 1/6** and **0 < q**_t_ **< 1**, when **δ > 0** it comes out that **q’ < q**_t_, so that as **t** tends to infinity **q**_t_ tends to zero and at equilibrium the proportion of males is **1/2**, corresponding to an **1 male : 1 female** sex-ratio. When **δ < 0**, however, it comes out that **q’ > q**_t_ so that the frequency of females will increase beyond 0.5. Extensive calculations using iteratively the formula above show that at equilibrium there will be an excess of females in the population and that in the range **-1/16 < δ < 0** approximately the allele **A** becomes entirely fixed (**q = 1**) in the population. With the exception of cases in which the modulus of **δ < 0** is **1/20** or less, the equilibrium frequencies of males and females will be approximately **1/3** and **2/3** (what corresponds roughly to a **1 male : 2 females** sex-ratio). In any case, the equilibrium value **q** is independent from the initial conditions and is a function of **δ** alone, as Figure 3 clearly shows. In this graph the curve for the equilibrium value **q** in the negative domain of **δ** was obtained by applying iteratively the recursion formula **q’ = f(q**_t_ **, δ)** or, when **-1/6 ≥ δ ≥ -1/16**, by using the numerical approximation (Bronstein et al., 2001) **q = a.Δ** ^b^ **.e** ^cΔ^ , where **Δ = |δ|**, with the coefficients **a = 0.098**, **b = -0.91** and **c = -3.32** obtained with the help of computer programs of non-linear regression analysis such as Sherrod’s *NLREG* software (2000).

When **δ > 0** (the case in which the X-linked mutation favors the production of males) a stable **1 male : 1 female** sex-ratio is attained, but the reverse situation (**δ < 0**) represents an important case in which the **1 male : 1 female** prediction fails. The situation is however not so drastic like the one in the **Y** allele case, since **|δ|** cannot have values larger than **1/6** and for most values of the frequency **|δ|** the population proportion of females would be at most twice that of males, what corresponds to a sex-ratio of **1 male : 2 females** approximately.

When **δ > 0, q’ < q**_t_, so the frequency of the mutant allele **A** (introduced by mutation and subjected to selection) is always very small, and the evolution of its frequency can be approximated with no significant loss of accuracy by the formula **q’ = q**_t_ **- 2δq**_t_ **(1-q**_t_ **)/3** , obtained directly from the complete formula by neglecting most squared and cubic terms of **q**_t_. For the case **δ < 0**, however, only the complete recursion formula should be used.

### Model 6: allele A, X-linked, that alters the sex-ratio, and that is located in a randomly inactivated locus

Similarly to the previous model, the offspring of the possible crossings at generation **t** has a proportion of males higher than 0.5, proportionally to the number of active **A** genes present in the parental pair. We shall have, therefore, six offspring types with a total of five different proportions of males and females (Table 4).

**Table 4 -:** Population frequencies of crossing types and progeny sex proportions. A is an X-linked mutant allele responsible for a distortion in the sex-ratio depending on the number of copies present in the parental pair. It is assumed that the (A, a) locus is randomly inactivated among females (model 6).

crossing types	crossing freq.	male proportion	female proportion
aa × a	P_t_(a).P_t_(aa)	0.5	0.5
aa × A	P_t_(A).P_t_(aa)	0.5 + δ	0.5 - δ
Aa × a	P_t_(a).P_t_(Aa)	0.5 + δ/2	0.5 - δ/2
Aa x A	P_t_(A).P_t_(Aa)	0.5 + 3δ/2	0.5 - 3δ/2
AA × a	P_t_(a).P_t_(AA)	0.5 + δ	0.5 - δ
AA × A	P_t_(A).P_t_(AA)	0.5 + 2δ	0.5 - 2δ

If we define, as above, **δ** as a positive quantity, its domain is obviously **0 ≤ δ ≤ 1/4**. But of course **δ** can be a negative quantity as well: in this case, its domain is **-1/4 ≤ δ ≤ 0**. In fact, when **δ = -1/4**, the five possible offspring male proportions listed on the table above take values 1/2, 3/8, 1/4, 1/8, and 0, respectively; when **δ = 1/4**, the corresponding values are 1/2, 5/8, 3/4, 7/8, and 1.

If we depart from an initial equilibrium population, that is, **P**_t_ **(a) ≈ p**_t_, **P**_t_ **(A) ≈ q**_t_, **P**_t_ **(aa) ≈ p**_t_^2^, **P**_t_ **(Aa) ≈ 2p**_t_ **q**_t_, **P**_t_ **(AA) ≈ q**_t_^2^, the frequency of males in the offspring generation **t+1** is given by

m_t+1_ = P_t_(a).P_t_(aa).1/2 + ... + P_t_(A).P_t_(AA).(1/2+2δ)

 = 1/2 + 2δq_t_ ,

while the frequency of females in this same generation (**t+1**) is given by

f_t+1_ = 1 - m_t+1_ = 1/2 - 2δq_t_ .

The genotype frequencies of **a** and **A** males and of **aa**, **Aa** and **AA** females in this generation **t+1** are obtained as in the previous model, taking the following values:

Pm_t+1_(a) = m_t+1_(a)/m_t+1_

 = [(1-q_t_)/2 + 3δq_t_(1-q_t_)/2]/(1/2 + 2δq_t_)

Pm_t+1_(A) = m_t+1_(A)/m_t+1_

 = [q_t_/2 + δq_t_(1+3q_t_)/2]/(1/2 + 2δq_t_)

Pf_t+1_(aa) = f_t+1_(aa)/f_t+1_

 = [(1-q_t_)^2^/2 - δq_t_(1-q_t_)^2^/2]/(1/2 - 2δq_t_)

Pf_t+1_(Aa) = f_t+1_(Aa)/f_t+1_

 = [q_t_(1-q_t_) - δq_t_(1-q_t_)(3+2q_t_)/2]/(1/2 - 2δq_t_)

Pf_t+1_(AA) = f_t+1_(AA)/f_t+1_

 = [q_t_^2^/2 - δq_t_^2^(3+q_t_)/2]/(1/2 - 2δq_t_) .

The allele **A** frequencies **Pm**_t+1_ **(A)** and **Pf**_t+1_ **(A)** among males and females are given by

Pm_t+1_(A) = m_t+1_(A)/m_t+1_

 = [q_t_/2 + δq_t_(1+3q_t_)/2]/(1/2 + 2δq_t_)

Pf_t+1_(A) = Pf_t+1_(Aa)/2 + Pf_t+1_(AA) 

 = [q_t_/2 - δq_t_(3+5q_t_)/4]/(1/2 - 2δq_t_),

so that, similarly to the previous case, the next generation frequency of the **A** allele in the whole population is given by

q’ = [Pm_t+1_(A)+2Pf_t+1_(A)]/3 

 = q_t_ - 2δq_t_(1-q_t_)(1+8δq_t_)/[3(1-16δ^2^q_t_^2^)] .

**Figure 4 -:**
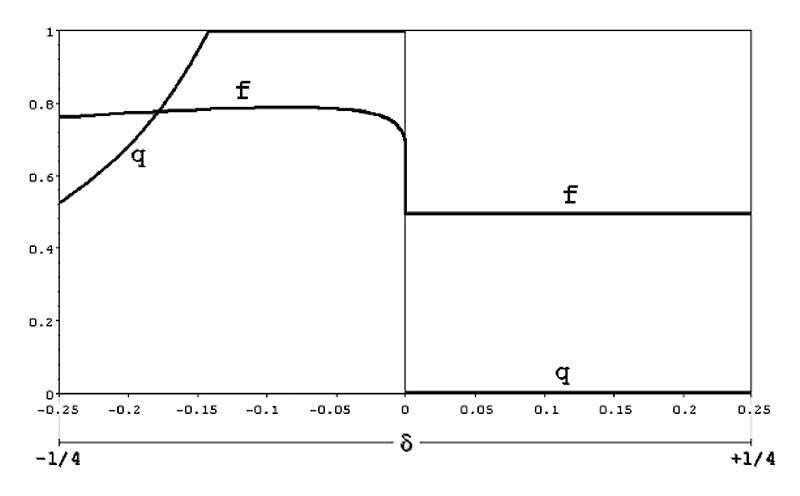
Population equilibrium frequencies of the mutant X-linked allele A (q) and of corresponding female frequencies (f), according to the value of the distortion factor **δ (model 6).**

Since **-1/4 < δ < 1/4** and **0 < q**_t_ **< 1**, when **δ > 0** it comes out that **q’ < q**_t_ , so that as **t** tends to infinity **q**_t_ tends to zero and at equilibrium the proportion of males is **1/2**, corresponding to a sex-ratio of **1 male : 1 female**. When **δ < 0**, however, it comes out that **q’ > q**_t_ so that the frequency of females will increase beyond 0.5. Extensive calculations using iteratively the formula above show that at equilibrium there will be an excess of females in the population and that for approximately **-1/7 < δ < 0** the allele **A** becomes entirely fixed (**q = 1**) in the population. With the exception of cases in which **|δ|** is **1/20** or less, at equilibrium the frequencies of males and females will be approximately **1/4** and **3/4** (what corresponds roughly to a sex-ratio of **1 male : 3 females**); this represents another important case in which the **1 male : 1 female** prediction fails. In any case, the equilibrium value **q** is independent from the initial conditions and is a function of **δ** alone, as Figure 4 clearly shows. In this graph the curve for the equilibrium value **q** in the negative domain of **δ** was obtained by applying iteratively the recursion formula **q’ = f(q**_t_ **, δ)** or, when **-1/4 ≥ δ ≥ -1/7**, by using the numerical approximation (Bronstein et al., 2001) **q = a.Δ** ^b^ **.e** ^cΔ^, where **Δ = |δ|**, with the coefficients **a = 0.22**, **b = -0.89** and **c = -1.34** obtained, as in the previous case, with the help of computer programs of non-linear regression analysis.

Even when **δ > 0** (the case in which the X-linked mutation favors the production of males) a stable **1 male : 1 female** sex-ratio is attained, but the reverse situation (**δ < 0**) represents another important case in which the **1 male : 1 female** prediction fails. This situation, which is analogous to the one found in the previous model (without X chromosome inactivation), is however not so crucial like the one in the **Y** allele case, since **|δ|** cannot have values larger than **1/4** and for most values of the frequency **|δ|** the population proportion of females would be at most three times that of males, what corresponds to a sex-ratio of **1 male : 3 females** approximately.

Just like the case **δ > 0** of the previous model **5**, the frequency of the mutant allele **A** is always very small, and the evolution of its frequency can be approximated with no significant loss of accuracy by the formula **q’ = q**_t_ **- 2δq**_t_ **(1-q**_t_ **)/3**, obtained from the last complete formula. So for small values of **q**_t_ and when **δ > 0,** the evolutionary behavior of the **A** allele frequency is practically the same in both models **5** and **6**. For the case **δ < 0**, however, the evolution of allele frequency **q**_t_ is different in both cases and only the complete recursion formula should be used.

The models just presented/reviewd point out that disruptions of the 1:1 sex-ratio do not evolve under autosomal inheritance, but can evolve with sex-chromosome inheritance. Moreover, most serious disruptions, even leading to extinction, are only likely with Y-linked inheritance. Most of these conclusions are not novel in the general context, but some details are new, such as the models with alleles having additive effects on the sex-ratio, regardless of their parental origin. In fact, unlike basic models with sex limitation and non-additive effects (Hamilton, 1967), X-linked models in which the shift on the usual 1:1 sex-ratio is due to the number of copies of the mutant allele present in the parental pair lead to extraordinary but apparently well-tolerated population equilibrium proportions of males and females varying from 1:1 to 1:2 or 1:3 respectively, depending on the value of the distortion factor and whether the mutant allele is randomly inactivated or not.
